# Design and Preliminary Evaluation of a Smart Orthotic Videogame Controller Dedicated to Children

**DOI:** 10.1007/s10439-026-04035-7

**Published:** 2026-02-23

**Authors:** Fabio Lazzari, Jacopo Romanò, Roberta Nossa, Sara Meloni, Lorenzo Garavaglia, Eleonora Diella, Matteo Valoriani, Francesca Fedeli, Matteo Porro, Emilia Biffi, Simone Pittaccio

**Affiliations:** 1 Institute of Condensed Matter Physics and Technologies for Energy (CNR-ICMATE), National Research Council of Italy, Lecco, Italy; 2 Scientific Institute, IRCCS Eugenio Medea, Bosisio Parini (LC), Italy; 3 Pediatric Physical Medicine & Rehabilitation Service, Fondazione IRCCS Ca’ Granda Ospedale Maggiore Policlinico, Milan, Italy; 4 Fifthingenium, Milan, Italy; 5 FightTheStroke Foundation, Milan, Italy

**Keywords:** Wearable orthotics, IMU sensors, Machine learning, Gesture classification, Wireless controller, Exergame-based rehabilitation

## Abstract

This paper describes the development, fabrication and testing of Playcuff, a wearable device designed to act as a videogame controller for children with motor disabilities, which also provides an orthotic action to improve the control of the upper limb. The aim of this device is to empower children with motor impairment and enable them to access and enjoy gaming despite their disabilities. The videogame controller function was achieved through on-board gesture classification using a two-tiered Fine Tree machine learning algorithm integrated into the device’s firmware. Based on features extracted from two inertial sensors present on the device, the classifier was trained to identify in real time 22 classes representing different postures and movements of forearm and wrist, showing an accuracy higher than 94%. A cohort of children (*n* = 19, aged 9.01 ± 1.95 years old) with neuromotor impairment involving the upper limb were enrolled to test the device. The acceptability and effectiveness of the device were evaluated through a specific questionnaire: the resulting answers were heavily skewed towards appreciation (80.5%) rather than criticism. The methods of classification were found to be simple and effective in controlling the game. In conclusion, Playcuff was shown to be a versatile and well-received orthotic controller, which could be used in future also for videogame-based rehabilitation.

## Introduction

Every child has a right to play [1] and to have natural social interactions with their peers. Children with motor impairments face several difficulties in participating to play because the way games are constructed or presented does not support or conform well to their abilities [2]. This is also true for videogames, whose interfaces are not functional for use by individuals with some disability [3].

As it happens for the games conceived for the typically developing children, the affordability of videogames for children with neurological deficits strongly depends on some general characteristics of the games, such as the topic, difficulty, and aesthetics [4]. In the presence of disability, however, other factors become relevant. Some are linked to sensory or cognitive impairment, such as the colour schemes, visibility of characters, and clarity of the objective and challenges [4]; others are related to the physical interface with the platform [5]. A recent study [6] has surveyed different dimensions of videogame usability by children with disability, and it concluded that those children generally manifest positive feelings when playing with videogames, especially with cooperative ones, even if they are not fully accessible. Amongst the most desirable improvements required by the interviewed children and families, several were related to the characteristics of the controller. In particular, controllers should not rely on small buttons or sticks, since they are not easily usable by children with neuromotor impairments; they should be compatible with bi‐manual tasks so that the healthy or less-impaired limb can support the affected one; they can recall daily-life objects (e.g. a steering wheel of a car, handlebar of a scooter, etc.), since they are intuitive to use and facilitate disabled children, but the similarity to real objects should be balanced with the ease of use in order to avoid frustration; they should allow training functional, purposeful movements; they should be compatible with different platforms/consoles.

A number of solutions have been proposed for controllers that are alternative to classic joysticks, such as the Xbox Adaptive Controller (XAC, Microsoft Gaming, USA) or various types of steering wheels and pedals, microphones, gloves, touch screens and cameras [2, 7–9]. A smaller number is especially dedicated to gaming by children with motor impairments. Amongst the interesting studies and technologies in this group, many are described as implicitly or explicitly connected with possible uses in the field of rehabilitation.

There are many commercial devices that have been investigated as game controllers for rehabilitation purposes [10–16].

Devices such as the Wii controller can be used for occupational rehabilitative systems, but they can be uncomfortable, especially for small children or patients with difficulty using their hands [2]. VR systems for motion tracking like Kinect and Leap Motion Controller can be used to evaluate quantitative movements during a game session. However, they may not be reliable if vision conditions are not optimal or if assistive devices such as orthoses or wheelchairs are present [2].

Multisensor, multisegmental inertial measurement unit (IMU) sensor wearable systems can be used to obtain kinematic measurements of a part or the entire body and classify exercises execution or sport actions [17–21]. They have the drawback of requiring games developed *ad hoc,* are not easy to wear, and need complex calibration protocols [22] which is not always straightforward when dealing with children, even more so if they have disabilities.

For this reason, it may be reasonable to assume that controllers that focus only on a limited number of joint segments can facilitate practical use.

In the field of upper limb rehabilitation, what has always captured the attention of the scientific community is the articulation of the hand [10, 23]. There are numerous wearable gloves, both prototypal and commercial, developed with the aim of evaluating and classifying finger activities [24, 25]. However, these are often expensive and therefore poorly accessible devices [24]. Furthermore, the recognition of finger movements alone is limiting and may not be sufficient to effectively control games that do not only involve grasping objects.

To overcome this limitation, there are many strategies that try to make wearable gloves multisensors [25, 26], but often they are devices that are bulky and difficult to fit.

Wearable wristbands are less investigated in the literature than wearable gloves and may have the advantage of being non-invasive and easy to use [27].

For example, using a smartwatch, it is possible to classify gestures related to ping-pong [28].

Wearing two inertial measurement units (placed on the hand and forearm), it is possible through the use of machine learning to estimate the wrist posture. However, the features used as input must be chosen carefully. An example is shown in [29], where the maximum accuracy is low (less than 65%) and a performance loss is achieved when the user’s working posture changes significantly.

In [30], a good accuracy in estimating the range of motion (ROM) measure was obtained by comparing different machine learning algorithms. The correct estimate of the flexion–extension angle of the wrist, however, is conditioned by the presence of drift in the signals of the inertial sensors. Furthermore, difficulties in positioning the sensors were noted due to the small hands of some participants.

A multisensor composed of IMU and surface electromyography can be used to classify hand and wrist movements performed by adult stroke patients and control exergames [31].

Although the use of machine learning is certainly an interesting technique, if it is not implemented directly on the device, it requires specifically designed game systems and is therefore not very flexible.

Many of these works have demonstrated potentially useful systems and techniques for real-time control of games, but few have led to the creation of complete wearable devices ready to be used by children. In general, it is easy to verify in the literature that the attention to the construction aspect of the devices, the wearability and adaptability to the human body, are often overlooked. Also, aspects linked to the control of posture, which could be of help when dealing with neurological disorders, have only rarely been addressed.

A few wearable devices that combine orthotic actions with the control of videogames are actually known in the literature. Ates et al. [32] developed a passive orthosis designed for rehabilitation, also involving the use of serious games. Despite having some issues, such as an excessively restrictive wrist pronosupination, this device was well received in a preliminary evaluation with stroke patients. Interestingly, a following iteration of the same device [33] involving active elements was less successful: the authors conclude that complex, bulky and unfriendly looking devices have low acceptability for home use. This suggests that a light passive orthotic action might be preferable instead. Previous work [34–37] discusses the fundamental design features of dynamic upper limb orthoses for children, and the rationale of their application for postural control and enhanced feedback on motor execution in neuromotor disorders. Those concepts could be relevant in designing wearable controllers for videogaming and were applied in the present work.

Here we present Playcuff, a wearable device for children, which integrates two main functions: it is a wireless wearable controller for videogames, and exploits a dynamic orthotic action to support motor control.

Our device was designed keeping in mind user-centred methodologies [39, 40] with a specific focus on upper limb functionality, as we will further expand upon in the Methods. The design focus was on the flexibility of use, in terms of both usability for children with different impairments and interoperability with diverse consoles.

The final device is compact, compliant in its physical interaction with the user, and lightweight. It is produced in different adjustable sizes and has a low production cost. It could be employed prospectively also for game-based rehabilitation.

In the light of the literature analysis above, Playcuff’s combination of characteristics—which was already deemed desirable and yet to be attained by other authors [33]—is innovative with respect to the state-of-the-art.

The present paper describes in detail the technical implementation of Playcuff, laboratory tests, and a preliminary acceptability and usability assessment with children.

## Materials and Methods

### Device Concept and Technical Requirements

Playcuff has been conceptualised and designed to address the inclusion of children with motor disabilities. A multidisciplinary team comprising engineers, clinicians, therapists, and stakeholders helped define the population target and the requirements of the system during the GiocAbile project [38].

The target population is school-age children (e.g. 7–16 years old) with neuromotor disability affecting the upper limb. The considered deficits include arm and hand weakness, movement initiation and control. The clinical picture can include hemi- and tetra-plegia with spasticity, focal or generalised dystonia, dyskinesia, athetosis, and ataxia.

The purpose of the device is to allow natural and free interaction with the videogame, without imposing uncomfortable postures or movement limitations. The child should be put in the best conditions to try effective, multisegmental, functional gestures to control the action in response to exergame challenges. A mild dynamic stabilisation of wrist and hand, combined with an augmentation of proprioceptive feedback mediated by the orthosis mechanical response, is deemed useful to reduce co-contraction, support voluntary control, and contrast pathological patterns.

The system should be able to interpret complex gestures and translate them into simple data streams, allowing broad compatibility with different game platforms.

The device should support accessibility and inclusion of children in play, and we foresee that it ought to be applicable in medicalised rehabilitation settings too. Classification should thus include monitoring of the main submovements (along the primary functional degrees of freedom) and recognition of execution speed. Based on this information, it will be possible to conceive games implementing rather complex therapeutic schemes including variations in the precision and quality of the motor execution required to control the game effectively, as well as changes in goal difficulty, or impairment compensation. At this stage, the technical decisions made for the controller implementation should not interfere with the flexibility of future exergame design.

Table 1 summarises the main requirements that were set on the design and the corresponding specifications. These requirements were identified keeping in mind the user-centred design methodology applied to upper limb orthoses [39], and wearable devices for the control of exergames [40]. Additional requirements, i.e. the dynamic postural correction and the enhanced feedback [34], were added in order to address better the needs of our specific end users.

**Table 1:** Design requirements and specifications

Requirements	Specifications and choices
Ergonomics	
Wearability on the wrist and hand	Design on the general shape of a glove or wristband
Wearability on the left or right side	One ambidextrous design
Easy donning and doffing	No fingers in the glove
Comfort and effortlessness	Soft, limited weight, not too tight
Aesthetics	Possibility to change colour or decoration
Availability of different sizes	At least three sizes, and modular components
Free positioning of user in space	Wireless connectivity
Orthotic function	
Mild dynamic postural correction to maintain wrist and hand in neutral position*	Low-stiffness extensor springs, flexible shells, soft constraints, tunable personalised forces, …
Augmented feedback to support proprioception and motor control and decrease co-contraction	‘Dynamic touching’ [41] Viscoelastic customised response to movement [34]
Controller function	
Ability to capture forearm and wrist movements	Inertial measurement units (IMU)
Easy wireless connection at short distance and possibility to use multiple devices at the same time	Bluetooth^®^
Ability to classify overall upper-limb gestures	On-chip classification algorithm
Platform independence	Simple serial data transfer protocol
Sufficient autonomy (1 hours min.)	Rechargeable batteries
Versatility of the device	Possibility to control different exergames with a single device
Availability	
Low cost	Careful selection of materials and components

^*^Neutral position is the position for each joint in which the combined action of the muscle passive tension sums to zero moment

To identify user-centred requirements for videogame accessibility valid for our target population (see further) we used information collected through a survey [6], which was analysed and discussed jointly by a group of biomedical engineers, paediatricians, neuropsychologists, physiotherapists and representatives of patients’ family associations.

**Fig. 1:**
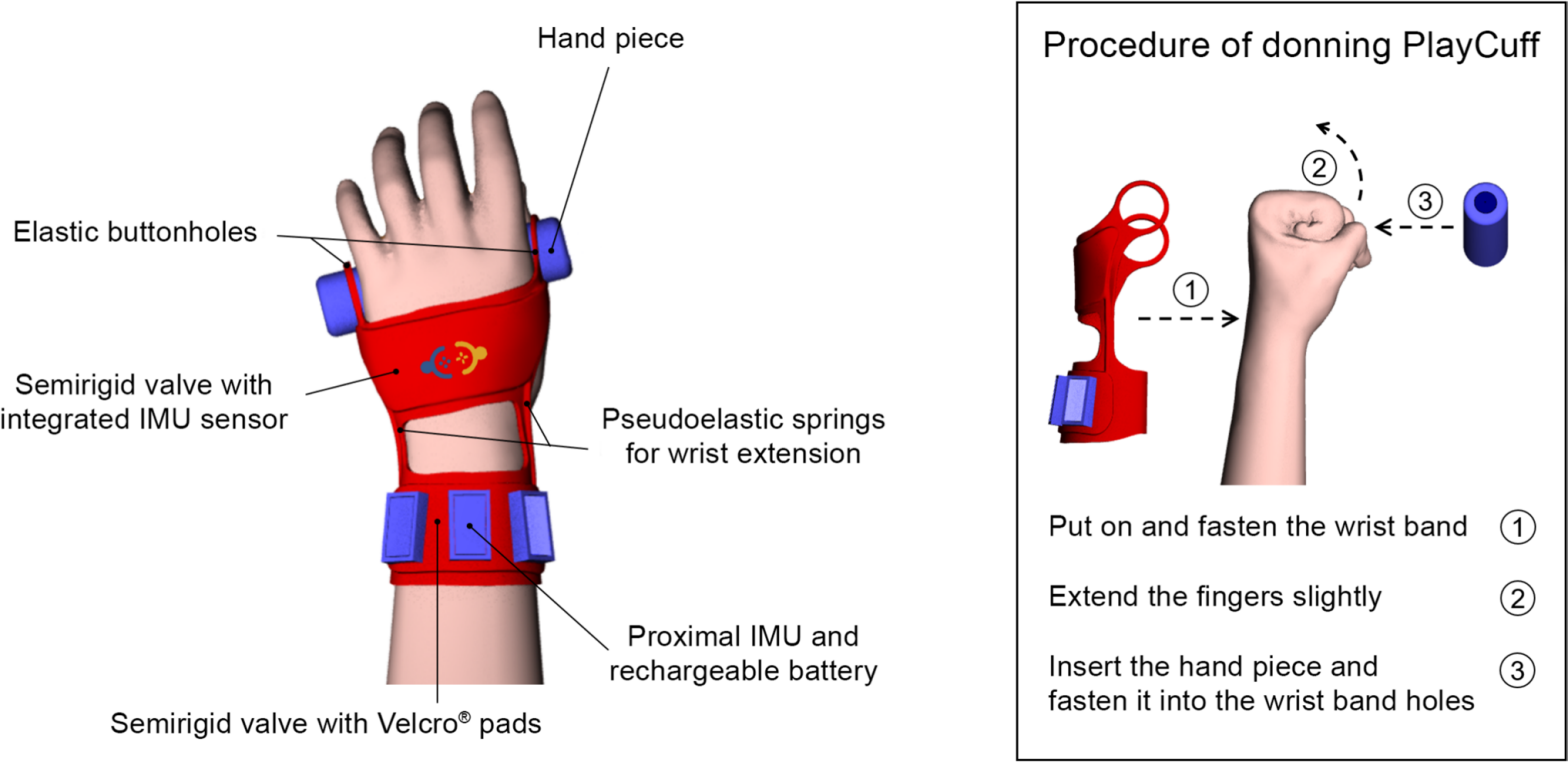
Concept of the wearable device, showing the main components (*left*) and the procedure of donning and doffing (*right*)

Based on these requirements, the concept was developed as in Figure 1.

### Technical Description: Device Design, Components, and Fabrication

**Fig. 2:**
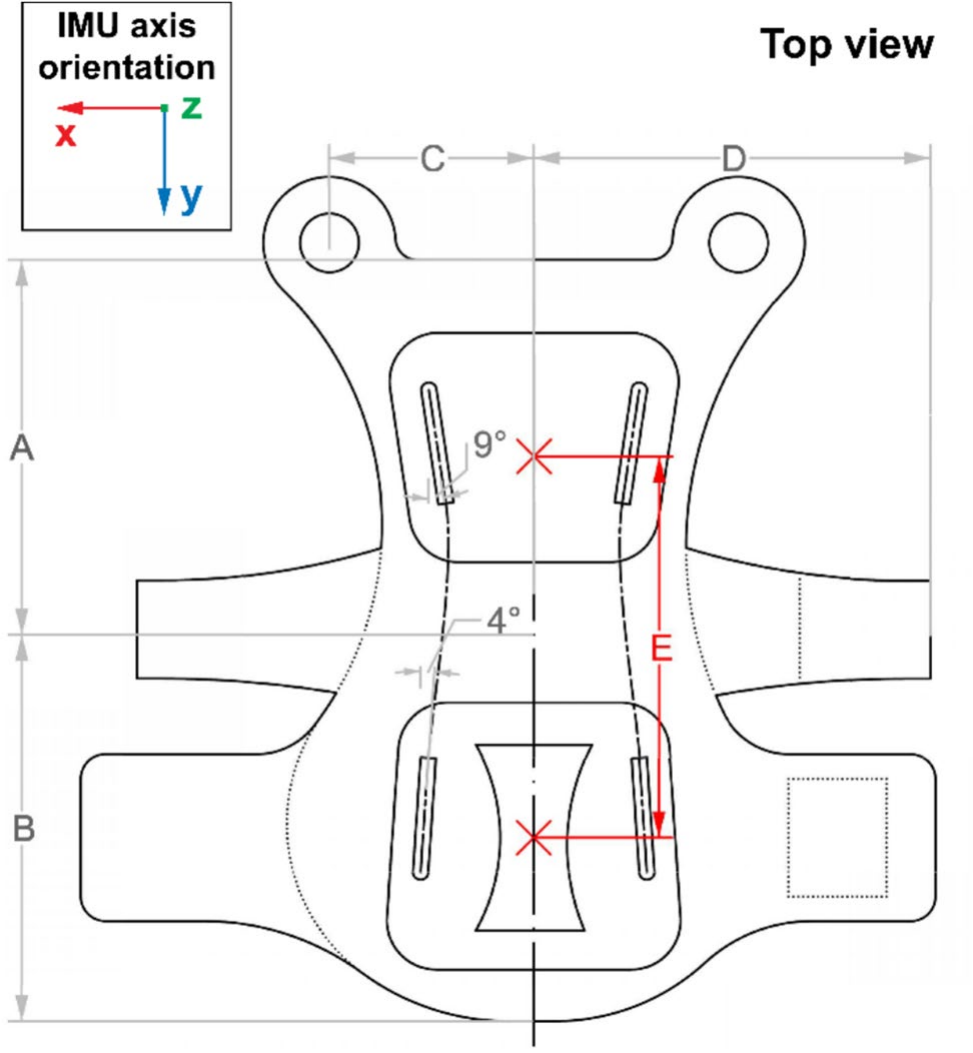
The paper pattern of the glove with some characteristic dimensions highlighted. The red crosses show the precise placement of the inertial units. The values of the dimensions are reported in the text for the three sizes of the device

The textile part of the device was made starting from paper patterns of three different sizes graded according to measurements provided by the participating families. In Fig. 2 is shown an example of the paper patterns with some characteristic dimensions that depend on the hand length (A), forearm length (B), hand width (C), and forearm circumference (D). For the three sizes made so far, these dimensions are, respectively, 80/74/31/95 mm (small), 83/82/37/105 mm (medium), and 86/110/52/141 mm (large).

The paper pattern is a single symmetrical planar shape including a forearm part with straps and a hand part with two open holes to the sides of the index and little finger, respectively. The device is fabricated by cutting and sewing together two layers of soft neoprene fabric. In the space between the two layers, different components can be inserted before finalising the stitching. Along the sides, two straight-annealed NiTi pseudoelastic alloy bars are laid in curved channels to generate the required orthotic action [34]. For the prototype, 1 mm diameter wires were selected, but the thickness can be changed to adjust rigidity for different patients (viz., hypertonia, pathological stretch reflex [35]). The curvature makes the wires somewhat stiffer under out-of-plane loading, so as to control wrist bending; the intrinsic pseudoelastic response produces low-modulus spring action, and also generates viscoelastic (dissipative) loads. The torque applied by the orthosis for fully extended wrist is of the order of 20 Ncm (measured according to method in [37]) irrespective of glove size, which can be felt but does not hinder movements.

Over the dorsal aspects of the hand and forearm, two semirigid shells made by fused filament 3D printing in thermoplastic polyurethane (TPU) are sewn in. They include cylindrical ducts to hold the NiTi wire ends and central pads to fix the IMU sensors (LSM9DS1, STMicroelectronics, Italy) (Figure 2). The two inertial units are soldered on boards and connected to the microcontroller unit (MCU, dsPIC33EP256MC202, Microchip, USA) by electric wires. The electronic board on the forearm includes in addition to the IMU and the MCU the Bluetooth^®^ transmitter (RN42-I/RM, Microchip, USA), and it is enclosed in a case, 3D-printed in poly(acrylonitrile-butadiene-styrene) co-polymer (ABS), alongside a LiPo rechargeable battery (3.7V 155mAh, LP-402025-1 s-3, BAK, China).

For the three different sizes, the distances between the IMU sensors were, respectively, 78 mm (small), 82 mm (medium), and 95 mm (large). The electric wires connecting the electronic boards were inserted between the fabric layers in parallel with the NiTi wires: the stiffer element (the metallic wire) was therefore the only one applying the desired force to the wrist during flexion–extension, and the thin electric wires did not affect the wrist movements to any significant degree.

Besides the main glove or cuff (in three sizes), the device includes a modular component (in three sizes as well), in the form of a solid ABS bar lined with a soft silicone (polydimethylsiloxane (PDMS)) rubber to interface the palmar aspect of the fingers. The bar can be slid into the hand (with some care if it is clenched into a spastic grip) and the two ends are then eased into the glove holes. That is sufficient to wrap fabric around the hand and hold it firmly in place. Donning is complete after securing the forearm part, which is done by passing the straps under and about and fastening the Velcro^®^ pads. This allows donning, wearing and doffing of the device without involving finger movements or imposing restrictions of use, with the aim of increasing the comfort and acceptability of the device.

It should be emphasised that the finger bars and gloves of different sizes can be paired interchangeably, adding to the device’s modularity and adaptability.

### Choice and Training of the Classifier

The classifier implementation was two-tiered, with a first tier identifying forearm movements and the second one taking care of wrist actions. The different forearm classes referred to eight different basic forearm gestures and 10 held positions. Each of these can be coupled with one of four wrist classes. To limit the variables and therefore the complexity of the model, the 8 static positions with the forearm horizontal were compacted into a single class (sH) before training the classifier and were re-separated in real time using the current pronosupination angle estimated from the accelerometer readings. All the classes are listed in Table 2. Speeds of forearm movement execution were recorded and grouped after the classification into three levels: slow (below 85 °/s), medium (between 85 and 170 °/s) and high speed (above 170 °/s).

**Table 2:** Movement classes of the forearm and wrist

Classes		Forearm classes (Tier 1)*
		*Held positions*
sH	sH_P	Static forearm in a horizontal range–thumb left
	sH_PN	Static forearm in a horizontal range–thumb 45° between left and up
	sH_N	Static forearm in a horizontal range–thumb up
	sH_SN	Static forearm in a horizontal range–thumb 45° between right and up
	sH_S	Static forearm in a horizontal range–thumb right
	sH_SM	Static forearm in a horizontal range–thumb 45° between right and down
	sH_M	Static forearm in a horizontal range–thumb down
	sH_PM	Static forearm in a horizontal range–thumb 45° between left and down
sU		Static forearm pointing up (*forearm directed above the horizontal range*)
sD		Static forearm pointing down (*forearm directed below the horizontal range*)
		*Active movements*
mU		Movement of raising up the forearm within the horizontal range
mD		Movement of lowering down the forearm within the horizontal range
mR		Movement of the forearm rightwards within the horizontal range
mL		Movement of the forearm leftwards within the horizontal range
dU		Movement in any direction with the forearm directed above the horizontal range
dD		Movement in any direction with the forearm directed below the horizontal range
rA_CW		Clockwise axial rotation of the forearm (*like turning door knob to open*)
rA_CCW		Counter-clockwise axial rotation of the forearm (*like turning door knob to close*)

Classes	Wrist classes (Tier 2)
sW	Still wrist
mWF	Wrist flexion movement
mWE	Wrist extension movement
errW	Any other wrist movement

^*^“Horizontal range” indicates any orientation, for which the forearm is directed at an angle between + 45° and − 45° relative to the horizontal plane.

All gestures described in Table 2 were repeated multiple times, by different healthy adults (see Section “Subject Enrolment and Usability Study Protocol”) whilst wearing the device. The participants were shown the gesture to be executed and asked to repeat it whilst varying at their will the speeds and exact trajectories. In particular, active movements of group (mU, mD, mR, mL) were repeated at 3 different velocities (low, medium, high, as interpreted by the subject), with 2 modes (twice precise and twice casual), and with 3 forearm postures (pronated, supinated, and neutral), for a total of 36 times each. The active movements of group (dU, dD) were carried out by moving the arm elevated above the head (or below the waist) in casual patterns for 10 s each. The active movements of the group (rA_CW, rA_CCW) were repeated at 3 different velocities with the shoulder elevated at 5 different heights from below the waist to above the head, for a total of 15 times each. The wrist movements (mWE, mWF) were carried out both isolated and coupled with movements of the group (mU, mD, mR, mL). In the first case, the shoulder was elevated at three different heights or the forearm was at three different pronations (9 combinations each); in the latter case the wrist was extending or flexing towards the direction the arm was moving (4 combinations each). The errW movement was repeated according to the same pattern as the other wrist movements by oscillating between radial and ulnar deviations. The held positions were retrieved from the intervals separating different movements of the preceding trials.

The measurements were performed in various sessions, alternating between two different devices and without calibrating the IMU inertial sensor at the beginning of the acquisition or at any other time.

To train the classifier, in addition to the input features derived from the IMU sensor signals, a list of output labels (expected or true classes) was required, which was created by assigning a class from Table 2 to each time instant. The collected data were first divided into various datasets containing the repetitions of single gestures. Subsequently, the moments in which the specific gesture was performed were distinguished from when the segment was still. Static forearm positions were associated with time instants in which the total rotation of the sensor was less than 30 °/s. The wrist was considered stationary when the difference between the rotations of the two sensors was less than 40 °/s.

All collected data were merged into a single dataset, containing just over 40,000 instants of time, and used to train a single, subject independent, two-layer classifier. These data were used only for the training phase, carried out using a supervised scheme with cross-validation. Matlab Classification Learner Toolbox (Mathworks, Natick, MA, USA) was used with a 5-fold cross-validation technique to train and optimise the performance of the models minimising the risks of overfitting.

Different classifier families were tried, such as decision trees [42], discriminant analysis [43], Naive Bayes [44], Support Vector Machine (SVM) [45], k-nearest neighbours (KNN) [46], and ensemble classifiers [47].

The final choice was made also considering that both tiers of the classifier should be run by the microcontroller unit (@59 MHz) firmware on the forearm board. The microcontroller clock frequency was chosen according to its power consumption, so as to meet the minimum requirement for battery duration. Another requirement was set that a gesture class should be sent to the game console at a rate of 20 Hz to enable smooth control and a seamless playing experience. The features were extracted at the same rate, by post-processing with a moving average filter the IMU data acquired at 200 Hz with a 10-sample window with equal weights. As a consequence, there was no overlap between the windows of two consecutive features so as to make them independent from the previous ones.

For the present application, an initial feature list including different combinations of IMU signals and derivatives was created including data from the accelerometer and gyroscope, whilst the magnetometer was not used. In fact, we considered its information, in the absence of a calibration, less significant, and less robust than that of the other two sensors. The initial list was then subjected to feature reduction (no feature transformation) aiming to maintain a high level of explained variance whilst decreasing model dimensionality. This process involved multiple training of all the classifier families considered with the same sets of input features, so as to select the one that showed the highest precision and which at the same time respected the integration requirements in the device firmware. Matlab Coder was employed to translate the trained classifiers into C language, so as to perform operational tests and subsequently integrate the final classifier into the microcontroller firmware.

The features selected at the end of this refinement process are summarised in Table 3. These are the three components of the acceleration and angular velocity for the forearm tier, whilst for the wrist tier we selected the absolute value of the difference in the angular velocities of the wrist and the forearm, the difference in absolute values of the same angular velocities, as well as the absolute value of the forearm angular velocity.

**Table 3:** Features used as inputs to the two-tiered classifier**

Input features to the forearm classifier (Tier 1)	
Feature 1	${a}_{X}^{F}$
Feature 2	${a}_{Y}^{F}$
Feature 3	${a}_{Z}^{F}$
Feature 4	${\omega }_{X}^{F}$
Feature 5	${\omega }_{Y}^{F}$
Feature 6	${\omega }_{Z}^{F}$

Input features to the wrist classifier (Tier 2)	
Feature 7	${\omega }_{X}^{W}-{\omega }_{X}^{F}$
Feature 8	${\omega }_{Z}^{W}-{\omega }_{Z}^{F}$
Feature 9	$\sqrt{{({\omega }_{X}^{F})}^{2}+{({\omega }_{Y}^{F})}^{2}+{({\omega }_{Z}^{F})}^{2}}$
Feature 10	$\sqrt{{({\omega }_{X}^{W})}^{2}+{({\omega }_{Y}^{W})}^{2}+{({\omega }_{Z}^{W})}^{2}}-\sqrt{{({\omega }_{X}^{F})}^{2}+{({\omega }_{Y}^{F})}^{2}+{({\omega }_{Z}^{F})}^{2}}$
Feature 11	$\sqrt{{\left({\omega }_{X}^{W}-{\omega }_{X}^{F}\right)}^{2}+{\left({\omega }_{Y}^{W}-{\omega }_{Y}^{F}\right)}^{2}+{\left({\omega }_{Z}^{W}-{\omega }_{Z}^{F}\right)}^{2}}$

^**^Where ${\mathrm{a}}_{\mathrm{i}}^{\mathrm{j}}$ are the accelerometer signals and ${\upomega }_{\mathrm{i}}^{\mathrm{j}}$ are the gyroscopic ones. Subscripts *i* = X, Y, Z referred to sensor axes (Y pointing along the body segment proximally and X to the left). Superscripts j = F, W referred to the forearm and wrist, respectively.

Amongst those tried, the best-performing classifier model, which met both execution speed and memory requirements, was found to be the Fine Tree algorithm. All training sessions performed with a smaller number of input features gave lower accuracy results than the selected set.

From the final verification of the speed requirements of the selected model, it emerged that the total time between reading the IMU registers and sending the classified on-board gestures via Bluetooth (Baud rate 115200) is less than 27 ms.

### Technical Tests

Following the estimation of classifier accuracy by means of the chosen cross-validation scheme, some additional technical tests were conducted to assess the model functionality. After integrating the definitive algorithm into the device, new measurements were performed. We focussed on verifying classification quality during real-time operation. To evaluate basic reliability during use, signals from the IMU sensors were acquired synchronised with the result of the classifier executed on-board. The same subjects enrolled to gather data for the training phase (see Section “Subject Enrolment and Usability Study Protocol”) carried out pre-established sequences of gestures corresponding to different classes. The true classes, assigned to each instant of time a posteriori based on the IMU sensor data according to the same rules used for the training phase, were compared timeframe by timeframe with the classes predicted at 20 Hz by the algorithm running on the microcontroller. We looked for long trains of misclassifications in adjacent timeframes that could hinder smooth game control.

### Connection with PC and Videogame Suite

The Xbox Adaptive Controller (XAC) was used to interface Playcuff with the PC running the videogames. The output of the classifier generated by the Playcuff firmware was translated to the XAC through an *ad hoc* Bluetooth^®^ bridging board. This device, which integrated a Raspberry Pi 4, after receiving data from Playcuff, converted it to the XAC codes corresponding to conventional joystick buttons and analogue sticks. The association between the Playcuff controller classes and the button combinations then used as input for the games was carried out via a browser configuration interface using the http protocol. The same was used to pair with the Playcuff device in use. Practically, the movements up, down, right, and left were mapped to the four directions of the XAC right stick and the speeds to the stick displacement, whilst forearm rotations were mapped to the X-axis of the left stick. The movements above and below the horizontal range were mapped to the A and B buttons, respectively. Wrist flexion was mapped to the X button and flexion to the Y button. Static poses were left unmapped, because in these games they all corresponded to no movement of the avatar.

The list of videogames, created specifically for this work, is reported in Table 4 together with the required actions. For each game, we attempted to select control gestures that were semantically tied to the in-game action.

**Table 4:** List of videogames tested by children during trials

Game title	Avatar actions	Playcuff classes
River in the jungle	Right/left movement	mR, mL
Fish for money	Forward/Backward	mU, mD
	Rotate	rA_CW, rA_CCW
Mining	Descend	mWF *or* mU
High-altitude flight	Right/left movement	rA_CW, rA_CCW
Ice Fishing	Up/down movement	mU, mD
	Right/left movement	mR, mL
Climbing	Up/down movement	dD, mD, mU, dU
Joining the crystals	Up/down movement	mU, mD
	Right/left movement	mR, mL
Platform	Forward/Backward	rA_CW, rA_CCW
	Jump	mWE *or* mU

Upon receiving a class, the XAC converts it to the relevant button or stick combination. The command acceptance method is implemented by continuously observing the last 6 received classes and accepting the command only if at least 3 of them correspond to the correct class for the current avatar action. Thus the method employs 0.3 s overlapping windows with a sampling rate of 20 Hz. This filters out classification noise, thus smoothing and making more robust the game control inputs even with respect to possible impairment-dependent motor uncertainties.

In case of discrete actions, such as jumping, accepting the command immediately translates into the corresponding action followed by a short interval of no acceptance (1 s). In case of actions where the avatar’s position evolves continuously, such as the movement of a boat on the screen, accepting the command translates into the time integration of its associated speed information (as transmitted via the sticks), i.e. a speed-dependent displacement increment.

### Subject Enrolment and Usability Study Protocol

For the acquisition of classifier training datasets and the real-time technical tests, 4 healthy adult volunteers (some of the authors) wore the controller and carried out the gestures. Healthy adults were selected to train the classifier, as opposed to healthy or disabled children, in order to ensure that the desired movements would be executed repeatably as prescribed. The volunteers signed informed consent to take part in the study.

Furthermore, 19 children (9.01 ± 1.95 years old, 12 male, 8 playing with the right hand, all playing with the most impaired limb), with motor impairment affecting the upper limb, were enrolled for an acceptability and functionality multicentre trial.

Inclusion criteria were: neuromotor disabilities and motor coordination disorders; age between 5 and 10 years; level of motor and cognitive functioning with International Classification of Functioning, Disability, and Health (ICF) [48] global score 1 to 3; Gross Motor Function Classification System (GMFCS) [49] I to IV; and Manual Ability Classification System (MACS) [50] I to IV. Exclusion criteria included seizures.

The patients had the following group characteristics upon enrolment. Median (interquartile value) scores for GMFCS and MACS were 2 (1.5) and 2 (1.8), respectively. Median (interquartile value) scores for cognitive and motor ICF were 1 (1) and 1.5 (1.8), respectively. The underlying condition was cerebral palsy for all patients.

Additionally, the families were administered the Assistance to Participate Scale (APS) [51] to evaluate the child’s participation in play and social activities.

The overall results of the APS settled on 4 (1). From these data, the typical patient showed moderate ability to walk and manipulate objects, variable global cognitive and motor functionality, and capacity to take part in activities with supervision.

The children’s families were first asked to provide measurements of their child’s hands. With a suitable subdivision into groups dimension by dimension, the measurements collected formed a base to draw the paper patterns, so that three glove sizes would be enough to fit reasonably well on all the hands.

After the devices were fabricated and passed the technical tests, the children were invited to try the controller on some of the dedicated games. Each child played at will using the wearable controller. They tested all the games discussed in Section “Connection with PC and Videogame Suite”.

After the play, children’s impression of the gaming experience were investigated by administering a customised questionnaire, which consisted of 7 sentences addressing 7 constructs (i.e. usability, control, enjoyment, disappointment, complexity, discomfort, and usefulness) scored using a five-grade Likert scale (Table 5). The results of the questionnaire were statistically analysed to extract the median and interquartile range of the obtained scores.

**Table 5:** Results of the questionnaire: number of answers received in agreement or disagreement with the proposed statements

	Statements	1	2	3	4	5	NA
1. Usability	“I was able to use the wristband to play games.”	0	1	1	8	9	0
2. Control	“I could get the wristband to do whatever I wanted.”	1	1	1	7	5	4
3. Enjoyment	“I found the use of the wristband fun.”	1	3	0	2	12	1
4. Disappointment	“I found the use of the wristband disappointing.”	9	1	0	1	1	7
5. Complexity	“I found the use of the wristband difficult.”	9	1	0	5	1	3
6. Discomfort	“I found wearing the wristband uncomfortable.”	12	1	0	3	2	1
7. Usefulness	“Wearing the wristband was useful for me to be able to complete the game/to make fewer mistakes.”	2	0	1	4	11	1
	Overall scores	34	8	3	30	41	17

For scores rearranged according to the semantics, see Table 6

The administration of that questionnaire was intended as a means to collect Patient-Reported Outcomes (PROs) according to the user-centred design approach and was especially instrumental in validating the effectiveness of the device. As the commonly used PROs measures, like QUEST [52] and OPUS [53], have been noted sometimes to be too generic [39]—and this was deemed the case for our device as well—we developed a new *ad hoc* questionnaire to evaluate the acceptability and effectiveness of the specific features of our device specifically for this pilot assessment.

The children and families signed informed consent to take part in the study.

## Results

### Fabrication of the Device

**Fig. 3:**
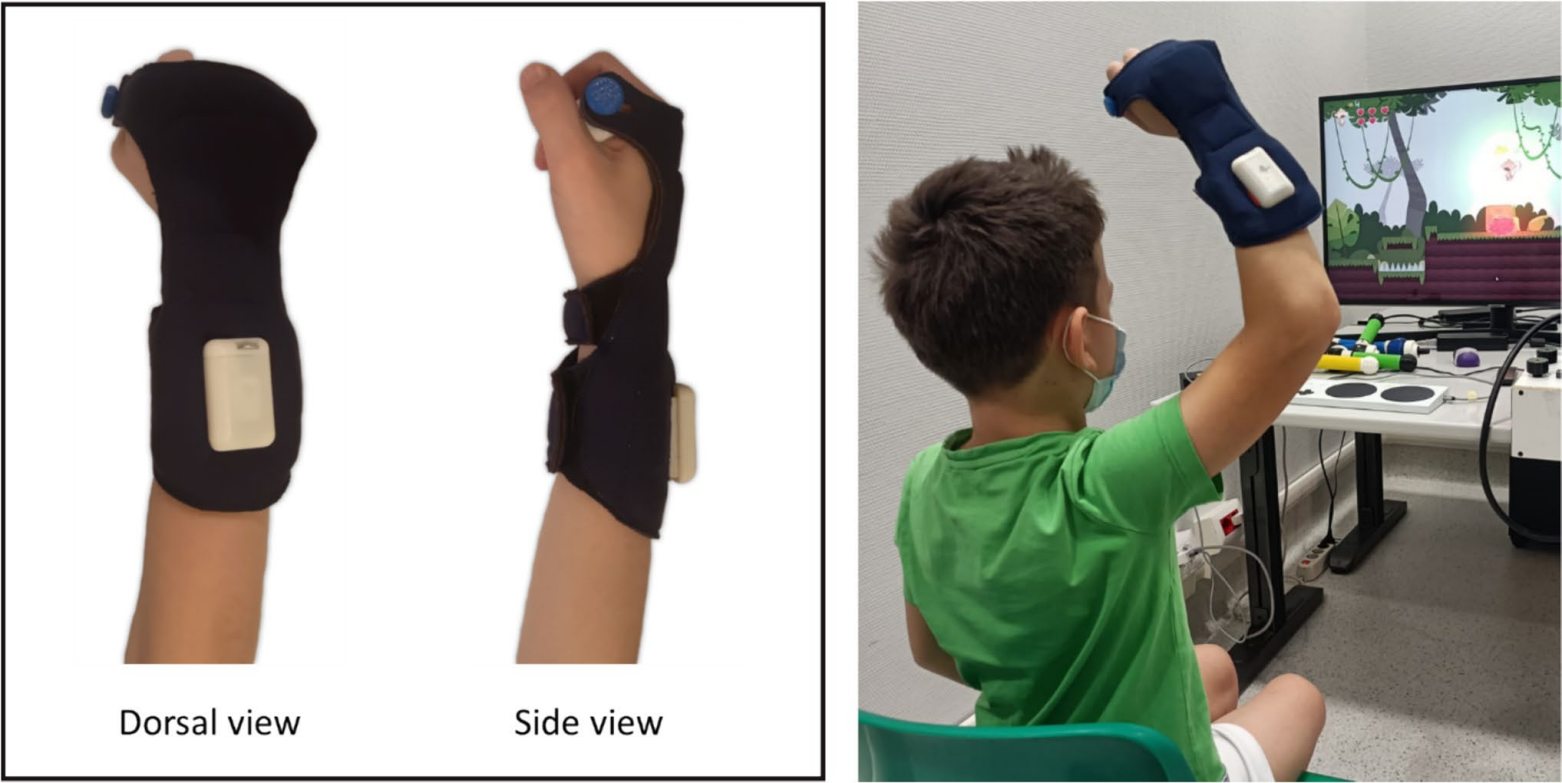
The device was fabricated in three sizes for the glove and three for the finger bar. The pictures on the *left* show dorsal and lateral views of the largest size. The box with battery and main electronic board is visible on the forearm, the hand holds the finger bar, whilst the second sensor on the hand dorsum and the NiTi elements on the sides of the wrist joint are not visible because they are embedded in the glove. For details on the placement of the components refer to Fig. 1. The photo on the *right* depicts one of the participants wearing the medium size during a play session

The device was fabricated according to the specifications. The result is displayed in Fig. 3. No problems occurred during the preparation of the components or the assembling phase. The device in three different sizes weighs 60 g (small), 80 g (medium), and 130 g (large). Preliminary use tests demonstrated that the batteries support continuous play for at least 2 hours. The cost for one device (singly produced) was around 180 €.

### Classification Performance

A Fine Tree algorithm was used for both classifiers. Matlab Classification Learner Toolbox allowed both to train and evaluate the performance of the two classifiers.

Other classifier families gave higher precision results than the selected ones but did not meet the calculation speed or memory requirements and therefore were not practically implementable in the device hardware. Regarding the forearm tier, best results for every family were obtained with Bagged Trees (ensemble classifiers, 98.6%), Cubic SVM (Support Vector Machine, 98.5%), Fine KNN (k-nearest neighbours, 98%), and Quadratic discriminant (discriminant analysis, 94.8%). The wrist tier achieved results of 99.9% and 99.8% with Quadratic SVM and Bagged Trees, respectively.

**Fig. 4:**
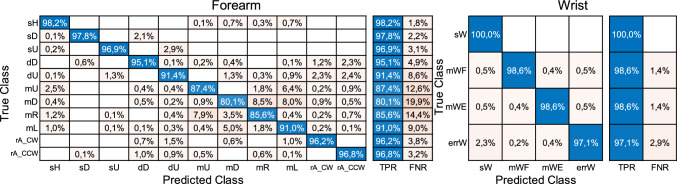
Confusion matrices of all the classification results obtained applying to the test subset the algorithms trained using the training subset. The overall accuracy was 94% for the forearm tier (*left*) and 99.5% for the wrist tier (*right*). A detailed description of all the classes can be found in Table 2. TPR is the true-positive rate, whilst FNR is the false-negative rate

The results on classification accuracy of the selected algorithm are presented in the form of confusion matrices (Figure 4), showing an overall accuracy of 94% for the forearm tier and 99.5% for the wrist one.

The forearm classifier was consistently able to distinguish held positions from active movements. Errors in differentiating between these two situations are limited and concentrated between groups of classes that correspond to similar spatial orientations. In particular, it can be seen that the sU class is slightly confused with dU (i.e. both with the forearm facing upwards), the sD class with dD (i.e. both with the forearm facing downwards), and the sH class with the classes mU, mD, mR, and mL (i.e. those with forearm between + 45° and − 45° above or below the horizon). These last four classes suffer the highest recognition errors, maintaining a True-Positive Rate (TPR) between 80.1% and 91%. Most of the confusion occurs amongst the four classes themselves, where rightwards and leftwards forearm movements are interchanged with upwards and downwards movements and *vice versa*.

The recognition of forearm movements performed beyond + 45° above the horizon (dU) and those performed beyond − 45° below the horizon (dD) is consistent: they are not mutually confused and either class is rarely exchanged with other classes representing movement situations, only seldom with those related to forearm axial rotation. The recognition error is mutual, in fact also rA_CW and rA_CCW are sometimes mistaken for dU and dD. The accuracy of forearm axial rotation recognition is however high, with a True-Positive Rate (TPR) greater than 96%.

The wrist action classification algorithm is very accurate in distinguishing all classes. The individual wrist flexion and extension movements, which in our case were the gestures used to control the game and therefore of utmost interest, were both recognised with an accuracy of 98.6%.

This initial assessment of the classifier done offline was complemented by the additional technical tests to evaluate its functionality also in real time. No significant differences in the performance of devices of different sizes were found in either of these tests.

In all real-time tests, the classifier correctly recognised the true gestures in the vast majority of timeframes. In particular, no examples of long trains (> 4) of misclassifications were observed.

Some representative examples of the forearm measurements are reported next. For symbols referring to the different classified gestures and positions, see Table 2 above.

**Figure 5.**
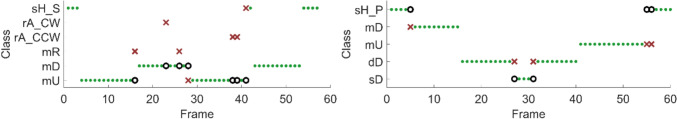
Frame-by-frame classification in two representative excerpts from repetitions of the shoulder adduction–abduction movement with different amplitudes, the elbow extended and the hand in a supine position (*left*) and in a pronated position (*right*). The solid green circles represent the correctly recognised classes. A red X represents an incorrect class predicted by the device, whilst the correct expected one is shown with a black circle

**Fig. 6:**
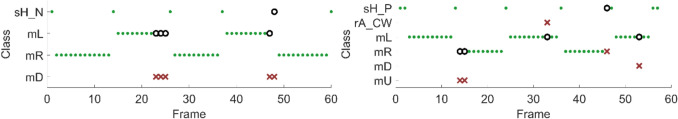
Frame-by-frame classification in two representative excerpts from repetitions of the shoulder flexion–extension movement, with the elbow extended and the hand in a neutral position (*left*) and in a pronated position (*right*). The solid green circles represent the correctly recognised classes. A red X represents an incorrect class predicted by the device, whilst the correct expected one is shown with a black circle

Figure 5 shows some repetitions of the shoulder adduction–abduction movement with different amplitudes, the elbow extended, and the hand in a supine (*left*) or pronated (*right*) position. Figure 6 shows continuous repetitions of the shoulder flexion–extension movement, with the elbow extended and the hand in a neutral position (*left*) or pronated (*right*).

Confirming what was observed by analysing the confusion matrix, in both figures it is possible to notice some prediction errors between classes that share similar regions of space (e.g. sD vs. dD) and only rarely classes that have no actual relationships (e.g. mU vs. rA_CCW). Most of the errors (about 80%) occur during transitions between states.

Overall, the errors are limited in number and do not compromise the quality of the recognition of the sequence of actions carried out.

**Fig. 7:**
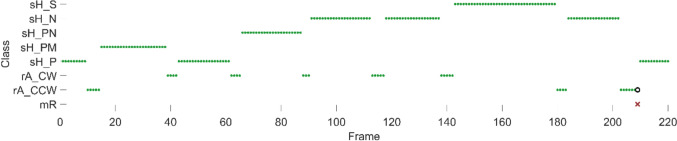
Frame-by-frame classification in one representative excerpt from repeated axial rotations of the forearm actions and static positions. Both classes are recognised with high accuracy. The solid green circles represent the correctly recognised classes. A red X represents an incorrect class predicted by the device, whilst the correct expected one is shown with a black circle

Finally, Figure 7 shows how the device is able to evaluate the posture of the forearm correctly during different axial rotations of the forearm (pronosupination).

### Acceptability and Usability

During preparation, it was always possible to find a suitable combination of glove and finger bar that would fit the children’s limb size adequately. No specific complications were encountered during donning or doffing of the device. Once the correct size for the patient was identified, it was very straightforward to obtain a correct alignment, both because the finger bar acts as a self-aligning element and because the position of the NiTi elements on the sides of the wrist makes a reliable reference.

The duration of the game sessions was, as expected, between 30 and 40 min. There were no drop-outs, and the interest was always maintained till the end of the allotted time (30 min).

The acceptability and usability were evaluated by a customised questionnaire administered directly to the children who used the controller to play videogames. Table 5 reports the given answers. In the table, the result “Disagree” includes levels 1 or 2, “Neutral” refers to level 3, whilst “Agree” combines levels 4 and 5. Considering the semantics, “Disagree” has a negative meaning for statements 1, 2, 3, and 7, whilst a positive one for statements 4, 5, and 6.

The recorded scores are non-equally distributed (*χ*^2^ test, *p < .001*). Rearranging the scores semantically, so that a positive opinion always corresponds to a higher numerical grade on the scale, the resulting distribution is found to be very skewed, with a weighted median corresponding to 5 and a weighted interquartile range of 1. Indeed, the overall ratio of appreciation vs. criticism sums up to 91 over 22, which means that 80.5 % of the expressed opinions were positive (Table 6). The device was regarded as usable in 83.1 % of cases, and it was found acceptable in 77.1 % of cases.

**Table 6:** Results of the questionnaire: number of answers received that correspond to positive, neutral or negative evaluations

	Statements	Criticism (grade 1 or 2)	Neutral (grade 3)	Appreciation (grade 4 or 5)
1	“I was able to use the wristband to play games”	1	1	17
2	“I could get the wristband to do whatever I wanted”	2	1	12
3	“I found the use of wristband fun”	4	0	14
4	“I found the use of wristband disappointing”	2	0	10
5	“I found the use of wristband difficult”	6	0	10
6	“I found wearing the wristband uncomfortable”	5	0	13
7	“Wearing the wristband was useful for me to be able to complete the game/to make fewer mistakes”	2	1	15
	Overall scores	22	3	91

For this representation it was necessary to rearrange the original levels taking the statement semantics into account (positive opinions correspond to higher grades of the scale)

## Discussion

### Validity of the Device Concept

As anticipated in the Methods section, the concept of the present device was based on several informed assumptions regarding the age, clinical conditions of the users and expected rehabilitation use in relation to gaming. The validity of the concept is difficult to parameterise. We assessed the usability and acceptance by children with different individual characteristics directly during play. The type of videogame varied from platformer to different arcade styles, in which avatars, ships, aircraft, or other objects had to be moved through the screen with the purpose to avoid obstacles and collect rewards. Each child could choose what game to play and for how long. The results of the customised questionnaire suggest that the device was well received. Questions related to usability, control, enjoyment, and usefulness were answered in most cases with appreciation, whilst discomfort, disappointment, and complexity constructs received low scores, confirming that the Playcuff was usable and well accepted. The general ergonomics can be confirmed. Our goal was to provide accessibility to gaming especially for children with movement disorders, and this objective guided design choices such as shaping the glove for easy donning and doffing, providing different targeted sizes, under-instrumenting by not including sensors on the fingers or upper arm to improve wearability and battery duration, the insertion of NiTi springs to provide mild orthotic action and functional stabilisation, and the choice of using sensors and a set of features for the classifier that work without requiring any initial calibration before playing. All these characteristics make our device quite unique, and thus different to compare to other smart gloves that can be found on the market with the purpose of controlling video games [24].

The choice to make the device wearable has as a consequence that holding or handling objects ceases being a mandatory and uncomfortable means to control the game, but can be made into a challenge to be rewarded in the game. The efficacy of such an approach could be at the basis of the appreciation demonstrated in the case of questionnaire constructs 2 and 7.

As explained above, a dynamic orthotic function is included out of neurophysiological considerations [34]: the repositioning and extension of the joints combined with enhanced proprioceptive feedback from the orthosis viscoelasticity might support improved motor control, especially in children displaying dyskinetic traits. With respect to previous work [34–37], in the present application we chose quite a thinner gauge (1 mm vs. 2 mm), because here the use of the orthosis was not intended as therapeutic but supportive of play, and we thought it had to be light and unobtrusive. Also, since for this study it was anticipated that several children with different manifestations and severity of motor impairment would use the device, personalised tuning of the wire force was not attempted. This would be easily achieved, as detailed in cited work, by changing the wire diameter, the alloy composition, the heat treatment or, dynamically by heating the wire, which would make the orthosis actively controllable. This function was not implemented to limit energy requirements, weight and bulk. The reported usefulness of the wristband in helping complete the game or to make fewer mistakes might be regarded as a positive effect of postural stabilisation.

### Controller Functionality

The classification scheme adopted for the present prototype was capable of supporting gaming. Both the predicted accuracy of the classifier and the overall performance in real time were very good in the laboratory tests with no observed instances of long misclassification trains that could undermine game controllability.

The alignment of the glove, which could have minor effects on the identification of held positions (especially if the glove is rotated around the forearm axis), was very easy to control and led to no measurable difference in the usability.

Some classification errors were found especially concentrated around transitions between two different classes. This happens because the training of the chosen classifier was focussed on recognising steady states (held positions or movement with quasi-constant angular velocities). During the transients, the movement-related acceleration that sums to the gravitational component is non-negligible and affects classification results. Since the effect is only limited to very few time instants, thanks to the command acceptance filter implemented in the videogame software as described in Section “Connection with PC and Videogame Suite”, it had no impact on the classifier usability: most of the transition errors are filtered out, and only the correct portions of the signal are used. The use of the threshold on minimum command repetition is also useful to avoid misclassification errors for classes mU, mD, mR and mL, which are generally used for moving avatars/objects, and are also the classes most often confused. Thanks to this, instances where avatars/objects would move randomly, against the player’s wishes, were exceedingly rare, and never resulted in failure in-game operation or were even perceived by the users.

Another advantage of the chosen classification algorithm, which makes it very robust, is that it is based on rather high thresholds on speed to separate static positions from movement. This choice, coupled with the absence of time integration (e.g. linked to sensor-fusion tasks) makes the device resilient to the effects of gyro sensor drifts and suboptimal calibration. In fact, the output of the classifier, even when used for several hours continuously, remained coherent.

The exclusion of the magnetometer signals from the chosen features also made the device immune to magnetic disturbances. This makes Playcuff a quick and easy device to use.

These results transferred quite well to the final scenario of actual use by paediatric patients. Indeed, the usability was deemed excellent by the interviewed volunteer users.

The possibility of including a calibration of the IMU was carefully considered, because in principle it could minimise classification errors at boundaries between rest and movement (cf. Section “Classification Performance”). On the other hand, the gain in precision would be very limited with respect to the high acceptance thresholds set in the game logic. So, the ability to continue playing without having to calibrate frequently (especially to catch up with gyroscope drift) was valued more than eliminating minor discrepancies which have no practical consequences during play.

### Compatibility with Videogames

From the point of view of data handling, the controller integrates movement classifiers that filter inertial sensor data on board. This choice should add to the device’s flexibility because simple numeric movement codes could then be mapped to common inputs of any console (buttons or analogue sticks), so that the spectrum of playable games is not reduced from the start by the necessity for the game logic to interpret exotic data structures.

The interfacing system (Bridge + XAC) has proven to be effective in translating the signals coming from Playcuff and making them available to the game. Technically, it was possible to verify that the time between the start of sending codes by Playcuff on the Bluetooth and the instant of complete reception of the commands by the game is always less than 2 ms. The general robustness is also confirmed by the fact that during the tests, with the device less than 5 m away from the bridging board, no transmitted data packet was lost. The advantages of XAC are the broad interoperability with different platforms. The downside, for our device, is the limited number of “buttons” (or channels) that the classes can be mapped to. Classic controllers typically have 14 digital buttons and 4 analogic axes, whilst the XAC can be used to map only 8 buttons and the 4 analogic axes. Whilst combinations of channels can be used to expand the usable number of identifiers of gestures, different channels cannot actually be used as combinations if the information they carry when used singly has to be transferred simultaneously and interpreted independently in certain circumstances.

Compatibility was hence tested also using different connectivity interfaces, e.g. by directly sending the same data stream to the Bluetooth^®^ port of a personal computer. This provides amplest possibility to map transmitted bytes to game actions. Clearly, commercial games would generally only recognise and use codes compatible with the ordinary joystick channels or channel combinations.

The method of classification and transmission was found to be simple and flexible and could be confirmed for future implementations of Playcuff.

### Space for Improvement

There are some directions for improvement that can be suggested in different domains.

*Aesthetics*. The current prototypes look quite plain, as no attempts were made to personalise the appearance, decorate the surface, or add accessories attached to the finger bar to simulate tools (magic wand, fishing rod, sword, etc.). The addition of these features would be quite straightforward and could improve the acceptability of the device.

*Classification* Whilst the current version of the classifier was suitable to control the tested games, improvements in this sense could be achieved by providing dedicated classifiers for players using the left or the right hand (to be selected in the game). This might increase specificity. We will also evaluate whether to introduce a new “transition” class to identify the states in which the current classifier most often returns incorrect recognitions.

Another development would consist in separating the classification of the rotation of the forearm along its axis from the other forearm movements. This would imply devising a third tier with a dedicated classifier (very simple, based mainly on the observation of a gyroscopic axis). With the possibility to map classes to a sufficient number of channels (e.g. compatible with the classic controller rather than the XAC), detailed (clinically relevant) data about that degree of freedom could be made available independently of other pieces of information.

The current classifier is based on training done with adult participants. The movements required were standardised but spanned different modes and speed of execution in order to increase the possibility to match gesture idiosyncrasies, including children’s movements. However, it would be interesting to add a paediatric cohort, whose typical way of execution might be subtly different due to the incomplete neuromotor development and characterised by somewhat different ranges for kinematic quantities such as the linear accelerations.

### Prospective Uses for Rehabilitation

The use of exergames aimed at the neuromotor rehabilitation of children is not new [54]. The rehabilitation of children with neuromotor disorders, however, remains a particularly complex issue, and so is the evaluation of their state and evolution during treatment. Practical difficulties descend from the complex interplay between the extremely varied disorder aetiologies and presentation and the underlying physical and mental development, which is also rapidly changing because of natural growth and condition evolution [55]. Additionally, the empowerment of paediatric patients, as well as their adherence to the therapy, is a matter of peculiar concern, especially if it is wished to involve them directly in decision-making.

The scientific literature describes a number of medical, physiotherapeutic, orthotic and robotic therapies that are applicable, depending on the clinical picture. Some (few) [56] of them were specially developed for the developmental age. On the other hand, several clinical scales and outcome measures have been specifically validated for the assessment of the upper limb in the paediatric population [57, 58].

In most dedicated approaches, the inclusion of play or, at least, playful settings in the rehabilitation programmes has already been recognised as a potent driver of children’s interest, helping them engage more fully with therapeutic exercises [59, 60]. The positive effect of adopting cognitively engaging and possibly gamified tasks has been verified also for older age groups [31, 61–63] and is gradually becoming a common specification for the development of new robotic or technologically based therapies even for the adult and elderly population.

Considering especially these devices, only a very limited number of biorobots and technologies [64] have been designed for children. This may be due to the overall scarcer number of expected users and to the added complexity of addressing the extreme size variability with adaptable or modular interfaces. Other critical aspects are linked to acceptability and usability, whose requirements are quite different for different age groups, due to the considerable neuropsychological evolution occurring throughout childhood and adolescence.

The key features of Playcuff, which contribute to explain its high acceptability, are its inexpensiveness and its user-friendliness: the device can be easily donned and is ready to use without requiring calibrations or other preliminary operations. These features make it feasible to hypothesise its potential application for home use, and not just rehabilitation in a clinical setting. The movements and poses that can be classified through the system are all components of purposeful gestures carried out in daily life. So, their selective and especially synergistic elicitation by play could be exploited for goal-oriented rehabilitation in semi-immersive game environments. In the light of its specificity for children with motor impairments, its easy wearability, usability, and its integrated functions of dynamic orthosis and videogames controller, we anticipate Playcuff to be a valid dedicated tool for the technology-aided rehabilitation of patients in developmental age. The wireless connectivity of Playcuff also paves the way to whole-body exercises to be carried out not just sitting at a table, but also standing or walking freely in space.

### Conclusion

This study demonstrates the feasibility of a wearable wristband composed of a glove and a finger bar, combining dynamic orthotic and exergame controller functions.

The result is a lightweight and easy-to-wear device, suitable for school age children.

The potential of machine learning was exploited to develop an on-board firmware executed in real time, which through technical evaluations in the laboratory proved to be able to correctly classify the chosen gestures. The classifier outputs were mapped onto 8 buttons and the 4 analogue axes using an XAC. This feature makes it flexible and potentially usable with any game whose button functions can be reconfigured.

Playcuff functionality, usability and acceptability were confirmed by pre-clinical tests with paediatric patients suffering from motor deficits of diverse aetiologies.

Our study has some limitations. Firstly, our sample size did not allow us to investigate whether there was a correlation between the clinical profile of our users and their responses to our questionnaire. We therefore do not know yet if there are certain features of our devices that are liked/disliked by children depending on their specific motor or cognitive functionalities.

A second limitation lies in the fact that we cannot provide with our study a direct comparison of the usability of Playcuff with that of other controllers, when tested by the same users and on the same games. An indirect comparison with other controllers designed for users with motor deficits comes from an analysis of the literature, as was provided in the Introduction. In case future studies that compare this device with similar technologies are carried out, it is suggested that, besides ad hoc questionnaires of usability, some standardised scales like SUS [65] or QUEST [52] be added to allow direct cross-platform analyses. The present system is not based on any device that must be handled or be held in the hand, nor requires the use of fine hand or digit movements for control. It is neither reliant upon external sensors that might need calibration, nor employs unusual commands like facial expressions, but only exploits broad upper-limb gestures. The simplicity and compactness of the Playcuff design, the wearability, adjustable size, orthotic action, and robust classification scheme were specifically meant to overcome problems identified for other technologies and resulted in a high level of appreciation.

In future, improvements in the aesthetics and available accessories will be implemented. Possible developments in the classifier structure could make gesture identification even more specific and support the evaluation of single degrees of freedom of interest. Enrolling a cohort of children to acquire data for classifier training could help verify whether more age-specific information might change the gesture identification performance and could be used to verify adult-derived model’s generalisability. Furthermore, it will be a focus of further research to develop well-designed exergames that can exploit Playcuff as a preferred controller to construct entertaining and efficacious rehabilitation programmes for the young. Indeed, rehabilitation technologies to be used by children need to be designed in a dedicated way, to be able to adapt to growth, very varied clinical conditions, and evolving needs and expectations, besides the foreseen rehabilitation purpose. Specially designed play aids can serve exergame-based rehabilitation.

## Data Availability

Sharing of the datasets used to train and test the classification algorithm was not explicitly authorised by the involved volunteers. The firmware implemented in the device that supports the results of this study is freely available in [66] (10.5281/zenodo.14718882).
